# The genetics and screening of familial hypercholesterolaemia

**DOI:** 10.1186/s12929-016-0256-1

**Published:** 2016-04-16

**Authors:** Raymond Henderson, Maurice O’Kane, Victoria McGilligan, Steven Watterson

**Affiliations:** Northern Ireland Centre for Stratified Medicine, Ulster University, C-TRIC, Altnagelvin Hospital Campus, Derry, Co Londonderry, Northern Ireland BT47 6SB UK; Department of Clinical Chemistry, Altnagelvin Hospital, Western Health and Social Care Trust, Londonderry, Northern Ireland BT47 6SB UK

**Keywords:** Familial hypercholesterolaemia, FH, cascade screening, screening, cholesterol, universal screening, atherosclerosis, CVD, CHD

## Abstract

Familial Hypercholesterolaemia is an autosomal, dominant genetic disorder that leads to elevated blood cholesterol and a dramatically increased risk of atherosclerosis. It is perceived as a rare condition. However it affects 1 in 250 of the population globally, making it an important public health concern. In communities with founder effects, higher disease prevalences are observed.

We discuss the genetic basis of familial hypercholesterolaemia, examining the distribution of variants known to be associated with the condition across the exons of the genes *LDLR*, *ApoB*, *PCSK9* and *LDLRAP1*. We also discuss screening programmes for familial hypercholesterolaemia and their cost-effectiveness. Diagnosis typically occurs using one of the Dutch Lipid Clinic Network (DCLN), Simon Broome Register (SBR) or Make Early Diagnosis to Prevent Early Death (MEDPED) criteria, each of which requires a different set of patient data. New cases can be identified by screening the family members of an index case that has been identified as a result of referral to a lipid clinic in a process called cascade screening. Alternatively, universal screening may be used whereby a population is systematically screened.

It is currently significantly more cost effective to identify familial hypercholesterolaemia cases through cascade screening than universal screening. However, the cost of sequencing patient DNA has fallen dramatically in recent years and if the rate of progress continues, this may change.

## Background

Familial Hypercholesterolaemia (FH, OMIM #143890) is a common genetic cause of premature Coronary Heart Disease (CHD). It is an autosomal, dominant, inherited disorder of lipoprotein metabolism that results in a raised Low Density Lipoprotein Cholesterol (LDL-C) plasma concentration.

Heterozygous FH (HeFH) is the most common monogenic disorder, affecting 1 in 200–250, twice as high as previously thought [1], with a penetrance of greater than 90 % [2]. It is believed that there are 34 million FH cases worldwide [1, 3] and that less than 1 % of potential patients with FH have been identified in most countries [3].

If HeFH is left untreated, there is a significant likelihood of CHD onset prior to age 55 (men) and 60 (women). Half of all untreated HeFH men and 15 % of women will die of CHD-induced myocardial infarction (MI) before these ages [3, 4]. Homozygous FH (HoFH) is rare with an estimated global prevalence of 1/160,000–300,000 [5]. However, when left untreated, patients with HoFH can succumb to MI as teenagers [4] with one reported case of a 4 year old child dying from CHD-induced MI [6].

In certain populations, the frequency of heterozygous FH may be markedly higher than 1 in 200. When a population is descended from a small number of colonizing ancestors amongst whom the prevalence of the condition was high, a *founder effect* occurs. Such founder effects are thought to be responsible for the prevalence of FH associated variants amongst Finns, Icelanders, Christian Lebanese, Tunisians, Gujarati South African Indians, Ashkenazi Jews, South African Afrikaners and French Canadians [7] that is as high as 1 in 67 for Ashkenazi Jews. Homozygous FH (HoFH) has been recorded as ten-fold higher in founder populations, principally due to consanguineous marriages [8].

Hydroxymethylglutaryl coenzyme A (HMGCoA) reductase inhibitors (statins) [9–11] are now the first line treatment for HeFH and HoFH. Prior to their emergence, mortality rates resulting from CHD in FH patients were nearly 100-fold greater in young adults aged 20–39, and approximately 4-fold greater in patients aged 40–59 than background [12]. However, there exists potential for improvement in the current detection and management of FH. Of those diagnosed, it has been shown that currently only 10–25 % receive appropriate therapy [13].

Here, we review the genetics of FH and the efficacy of FH screening programmes. We suggest that the expansion of screening programmes has the potential to contribute significant economic and social benefit.

## Review

### The genetic basis of FH

Elevated cholesterol was first demonstrated as a major risk factor for CHD in 1961 [14]. Lipoproteins were subsequently identified as a factor in atherosclerosis [15] and they were classified into the following cholesterol-carrying types in order of increasing density: chylomicrons, very-low-density lipoprotein (VLDL), Low Density Lipoprotein (LDL), intermediate-density lipoprotein (IDL) and high-density lipoprotein (HDL). The regulation of cholesterol via the LDL-receptor (LDLR) pathway featuring receptor-mediated endocytosis was recognised as critical to atherosclerosis [16] and this facilitated the identification of genetic defects that cause malfunction of the LDL receptor as a major risk factor [17].

**Fig. 1:**
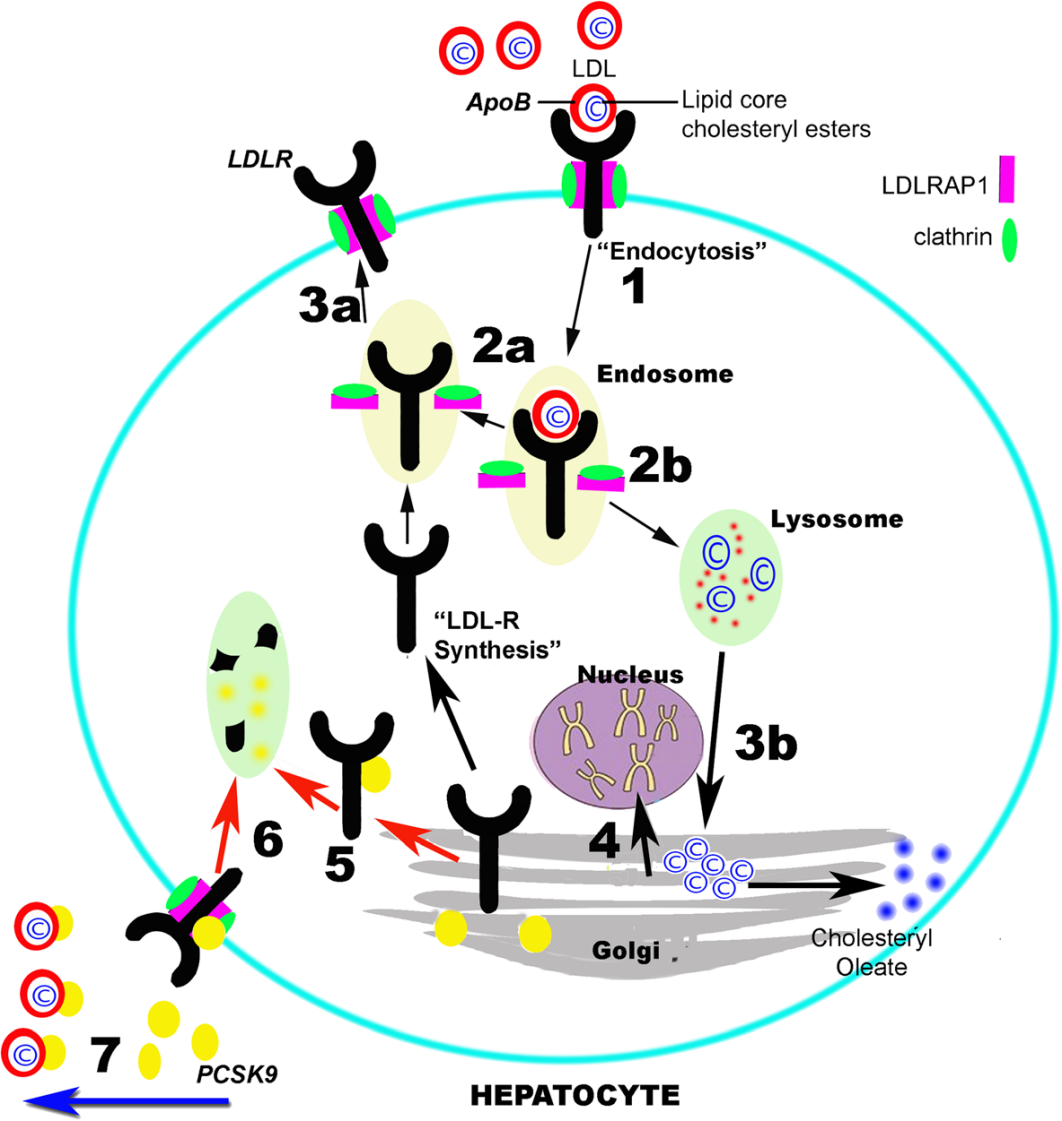
The LDLR pathway. The LDL receptor (LDLR), part of a LDLR/clathrin/LDLRAP1(ARH) vesicle, binds to the ApoB in LDL particles, internalsing them (1) [26]. The receptor-ligand complex dissociate and LDLR is either recycled (2a and 3a) or degraded (2b and 3b). Residual cholesterol levels regulate the transcription of LDLR (4). PCSK9 is endogenously secreted from the Golgi apparatus where it binds to LDLR (5) [93]. Alternatively, PCSK9 can exogenously bind to LDLR (6). Once internalised to the hepatocyte, PCSK9 directs bound LDLR to the lysosome for degradation. Recent evidence suggests that PCSK9 can bind to LDL via ApoB in free circulation (7) [94]

The majority of FH cases are caused by mutations in the *LDLR* gene, resulting in defective synthesis, assembly, transport, recycling or vesicle formation (Fig. 1). Mutations in the *LDLR* gene cause FH in 79 % of cases. Apolipoprotein B (*ApoB)* helps the LDL-receptor bind LDL and mutations in *ApoB* account for ~5 % of FH cases. Proprotein convertase subtilisin/kexin type 9 (*PCSK9*) degrades the LDL-receptor and gain of function mutations in PCSK9 account for <1 % of FH cases [18]. A very rare recessive form of FH is caused by mutations in low-density lipoprotein receptor adaptor protein 1 (*LDLRAP1*). The remaining 15 % of FH cases are either polygenic or are driven by monogenic mutations whose prevalence is not yet determined [18]. The latter include mutations in *APOE* [19], APOB [20], *SREBP2* [21] and *STAP1* [22].

The FH variant database maintained as part of the Leiden Open Variation Database (LOVD) stores the number of sequence variants for *LDLR*, *PCSK9*, and *LDLRAP1* [23–25]. Our discussion of the genetic basis of FH describes data from the current release of the LOVD. However, the number of reported variants and our understanding of their role is likely to develop as a result of future studies.

#### LDLR

**Fig. 2:**
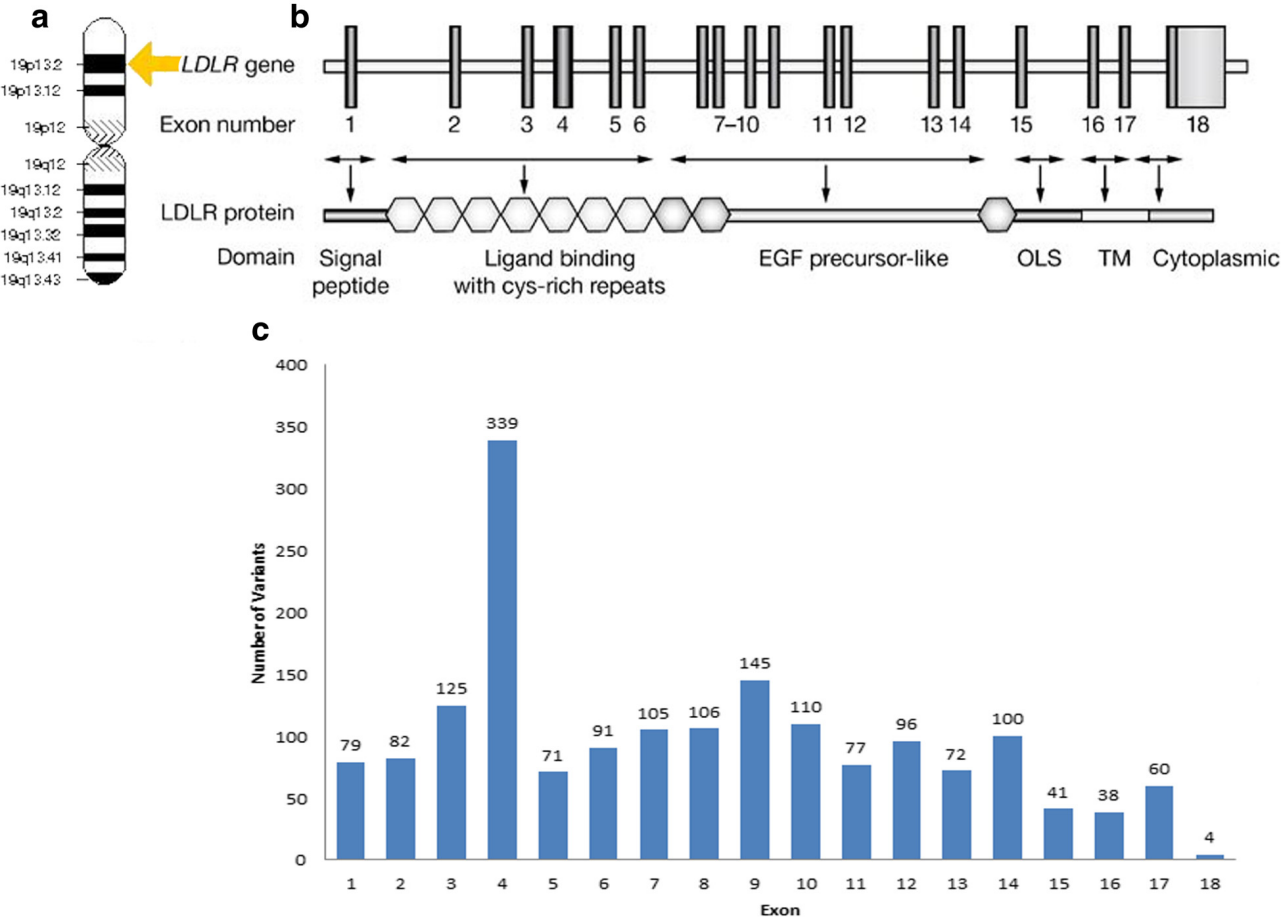
*LDLR* gene. **a.** Location of the *LDLR* gene, the short (p) arm of chromosome 19 at position 13.2. **b**. Numbered vertical bars represent exons, lone exons or sets of exons that encode the various domains of the LDLR protein. **c**. Currently 1,741 mutations have been identified in the exons of the *LDLR* gene. The phenotypic presentation of these sequence mutations are discussed in detail elsewhere [29, 30]. Partially adapted from [26]. Data extracted from [23–25]

To date research has uncovered a large number of mutations in the LDL-receptor protein associated with FH. The LOVD FH variant database describes 1741 mutations (retrieved 3rd July 2015). Of these, 1295 are understood to be unique variants, with 1064 predicted to be pathogenic, 143 predicted to be non-pathogenic, and 88 of unknown significance (personal communication, Dr. Sarah Leigh, 13 July 2015). Mutations can yield FH through a range of mechanisms. These include affecting splicing of the pre-messenger RNA (pre-mRNA), altering the promoter region that affects gene transcription, through single amino acid substitutions, creating premature stop codons and introducing large rearrangements. These mutations affect the structure and function of the LDL-receptor and range across the entirety of the *LDLR* gene (Fig. 2), with nearly all amino acid substitutions identified as having deleterious effects. When the mutation occurs as a large rearrangement, or in cysteine-rich repeats either as substitutions or premature stop codons, it produces an entirely non-functional protein [26]. Defects in a splice junction beside an exon may or may not effect splicing.

#### ApoB

**Fig. 3:**
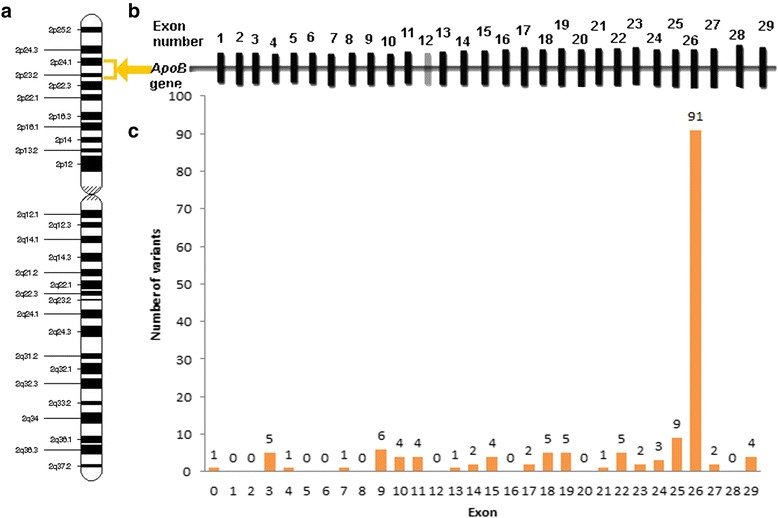
*ApoB* gene. **a.** Location of the *ApoB* gene on the short (p) arm of chromosome 2 between positions 24 and 23. **b**. Numbered vertical bars representing the exons. **c.** Only 8 disease-associated sequence variants have been found to occur in *ApoB*, and the majority of these are at the mutational hotspot exon 26. When translated this domain functions as the region for LDLR binding. Data extracted from [23–25]

*ApoB* variants are principally located on one exon, number 26 (Fig. 3). Patients with Familial Ligand-Defective Apolipoprotein B may have a milder form of presentation of FH than that caused by *LDLR* mutations [27].

#### PCSK9

**Fig. 4:**
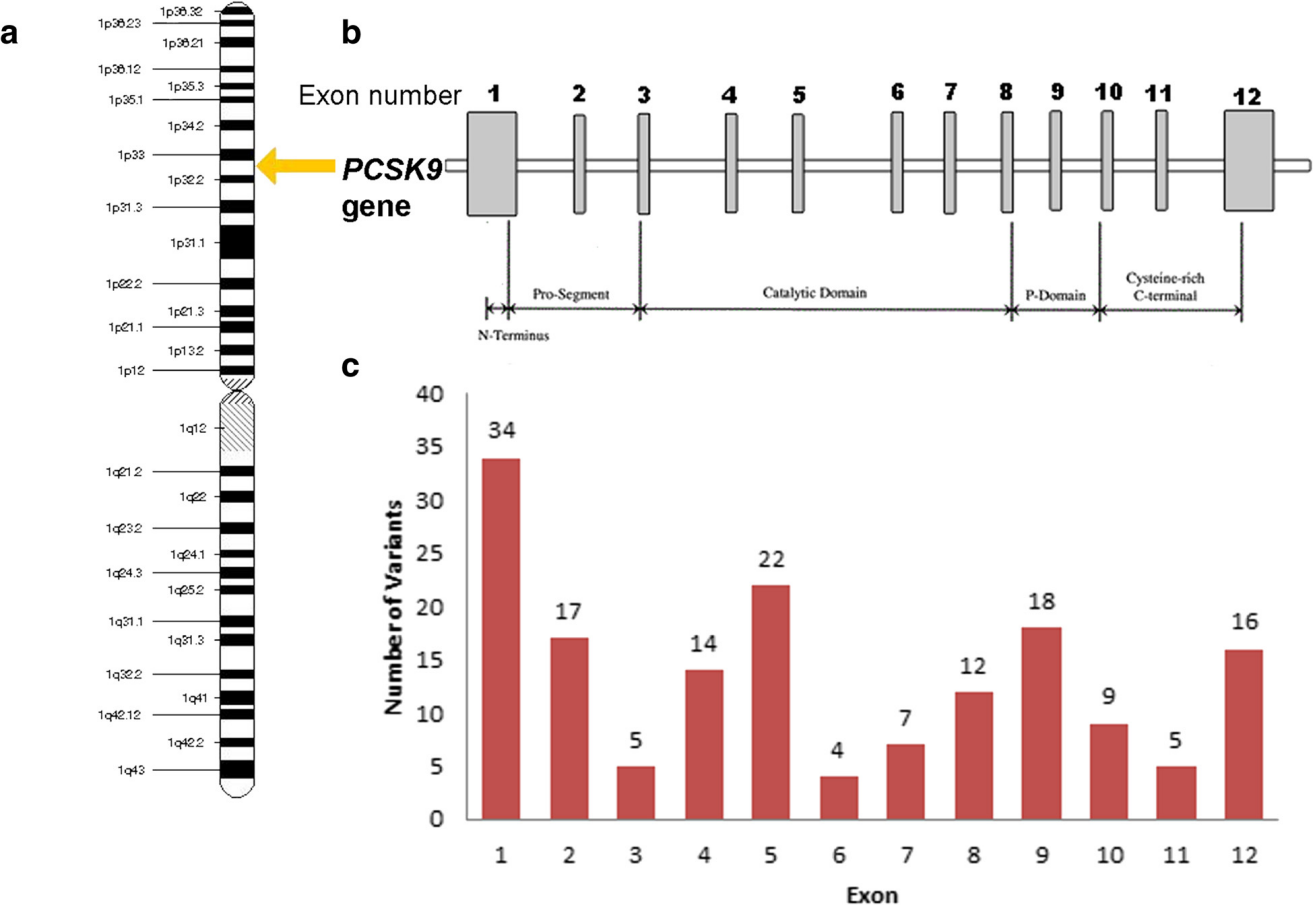
*PCSK9* gene. **a**. Location of the *PCSK9* gene on the short (p) arm of chromosome 1 at position 32.3. **b.** The numbered vertical bars represent exons. **c**. There are 163 mutations seen in the *PCSK9* gene, some causing gain-of–function and some causing loss-of-function. Data extracted from [23–25]

Missense mutations in *PCSK9* that cause a gain-of-function lead to a rare form of FH [28]. Loss-of-function in certain ethnic populations has been shown to result in lower LDL-C levels and protect against CHD [28]. Figure 4 shows both gain-of-function and loss-of-function variants along with variants of unknown significance [23–25, 29, 30].

*PCSK9* inhibition/repression has emerged as an important objective in clinical trials where *PCSK9* inhibitors have demonstrated significant cholesterol lowering efficacy [31].

#### LDLRAP1

**Fig. 5:**
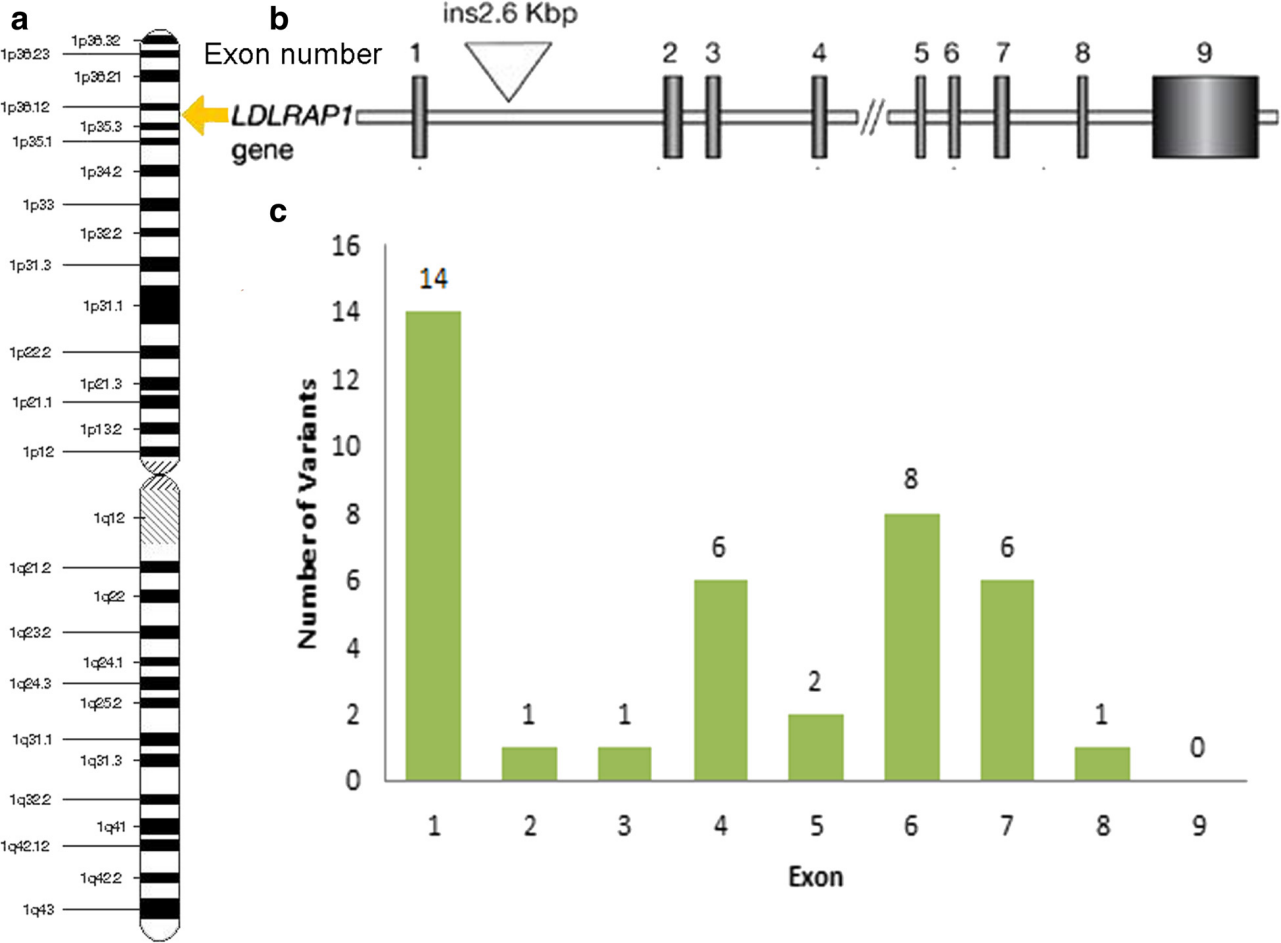
*LDLRAP1* gene. **a**. Location of the *LDLRAP1* gene on the short (p) arm of chromosome 1 at position 36.11. **b**. Numbered vertical bars represent the exons. **c**. 39 mutations seen in *LDLRAP1* and each exon mutation produces various phenotypic effects, for example, a mutation in exon 6 will be more responsive to lipid-lowering therapeutics [29, 30]. Data extracted from [23–25]. Partially adapted from [26]

LDLRAP1 mutations show a recessive model of inheritance. As such, this rarely-occurring disease is termed autosomal recessive hypercholesterolaemia (ARH, OMIM #603813) to differentiate it from the FH conditions attributable to *LDLR*, *PCSK9*, and *ApoB* mutations [32]. Figure 5 shows the distribution of known mutations across *LDLRAP1*. Exon 2 mutations lead to a phenotype similar to HoFH. Mutations in exon 6 have been shown to be more receptive to lipid-lowering therapy [29, 30]. ARH cases generally have lipid levels between those of HeFH and HoFH patients and ARH patients tend to be the progeny of ARH consanguineous marriages [33]. HDL levels are greater than those of patients with HoFH. Consequently, early-onset CHD is postponed. Unlike HoFH, no case of a patient under 20 years old with CHD has been recorded.

### Diagnosis

Identification of FH is primarily by clinical diagnosis with subsequent confirmation by genetic testing where possible. A family history of premature CHD, a clinical history of premature CHD, physical examination for xanthomas and corneal arcus and elevated plasma LDL-C concentration are all used in diagnosis. These characteristics have been used to develop the most widely used clinical criteria to aid diagnosis: the Dutch Lipid Clinic Network (DLCN) criteria, shown in Table 1 [34]. Genetic analysis is suggested if the DLCN score is >5. Two other widely used and clinically proven diagnostic tools are the Simon Broome Register (SBR) criteria used in the UK and shown in Table 2 [35] and the Make Early Diagnosis to Prevent Early Death (MEDPED) criteria used in the USA and shown in Table 3 [36]. No international standard currently exists. SBR and DLCN are similar in their choice of criteria, but SBR accepts the presence of a DNA mutation as a definitive confirmation of FH, whereas DLCN needs additional criteria for definite FH diagnosis. For patients with a diagnosis of ‘definite’ FH by SBR criteria, a causal mutation can be found in greater than 80 % of cases [7, 37]. Each system adopts different cholesterol cut-offs for concluding premature CHD. The MEDPED criteria rely on age-specific and family relative-specific total cholesterol (TC) only. Although easy to use, MEDPED does not incorporate clinical characteristics or identified FH gene mutations. Their relative merits have been explored in comparative studies [38] and, using a genetic diagnosis as the comparator, SBR criteria have demonstrated a sensitivity of 34 % and a specificity of 89 % for FH diagnosis [39].

**Table 1:** DLCN Diagnostic Criteria for FH

Group 1: Family History	Points
i. First-degree relative with premature CHD^a^	1
ii. First-degree relative with LDL-C > 95th percentile by age, gender for country	1
iii. First-degree relative with tendinous xanthomata and/or arcus cornealis	2
iv. Children under 18 years with LDL-C > 95^th^ percentile by age, gender for country	2
Group 2: Clinical History	Points
i. Premature CHD	2
ii. Premature cerebrovascular or peripheral vascular disease	1
Group 3: Physical Examination Points	
i. Tendinous xanthomata	6
ii. Arcus cornealis prior to 45 years	4
Group 4: LDL-C Levels	Points
i. LDL-C > 8.5 mmol/l (~330 mg/dl)	8
ii. LDL-C 6.5-8.4 mmol/l (~250-329 mg/dl)	5
iii. LDL-C 5.0-6.4 mmol/l (~190-249 mg/dl)	3
iv. LDL-C 4.0-4.9 mmol/l (~155-189 mg/dl)	1
Group 5: DNA Analysis Points	
i. Causative mutation in the LDLR, ApoB or PCSK9 gene	8
Total Score: -	
Definite FH > 8 points	
Probable FH: 6–8 points	
Possible FH: 3–5 points	
Unlikely FH: 0–2 points	
Genetic Testing For: -	
i. Patients with a score > 5 points	
ii. Patients with an obvious diagnosis of xanthomata with high cholesterol and a CHD family history	
Causative Mutation Found: -	
Genetic testing for all first degree relatives	

^a^CHD Before age 55 (men), 60 (women)

**Table 2:** Simon Broome Register Diagnostic Criteria

A diagnosis of explicit FH requires either (1), (2) or (3)	
1	i. Cholesterol higher than 7.5 mmol/L or LDL-cholesterol above 4.9 mmol/L in adult
	ii. Tendon xanthomas in patient or a 1st degree relative (parent, sibling, child), or in a 2nd degree relative (grand parent, uncle, aunt)
2	i. Cholesterol higher than 6.7 mmol/L or LDL-cholesterol above 4.0 mmol/L in a child under 16 years of age
	ii. Tendon xanthomas in patient or a 1st degree relative (parent, sibling, child), or in a 2nd degree relative (grand parent, uncle, aunt)
3	i. DNA based evidence of a functional LDLR, PCSK9 and APOB mutation
A diagnosis of probable FH requires either (1), (2) or (3)	
1	i. Cholesterol higher than 7.5 mmol/L or LDL-cholesterol above 4.9 mmol/L in adult
	ii. Family History of myocardial infarction (MI) before 50 years of age in a 2nd degree relative or below age 60 in a 1st degree relative
2	i. Cholesterol higher than 6.7 mmol/L or LDL-cholesterol above 4.0 mmol/L in a child under 16 years of age
	ii. Family History of myocardial infarction (MI) before 50 years of age in a 2nd degree relative or below age 60 in a 1st degree relative
3	i. A family history of raised total cholesterol - higher than 7.5 mmol/L in adult 1st or 2nd degree relative or higher than 6.7 mmol/L in a child or sibling aged under 16 years

**Table 3:** The US (MEDPED) Diagnostic Criteria for FH. FH is diagnosed if total cholesterol (TC) levels exceed the threshold stated [95]

Age (years)	First Degree relative with FH (TC, mmol/L)	Second Degree relative with FH (TC, mmol/L)	Third Degree relative with FH (TC, mmol/L)	General Population (TC, mmol/L)
<20	5.7	5.9	6.2	7
20-29	6.2	6.5	6.7	7.5
30-39	7	7.2	7.5	8.8
≥40	7.5	7.8	8	9.3

Recent research has developed a FH prognostic model, Familial Hypercholesterolaemia Case Ascertainment Tool (FAMCAT), composed of nine clinical factors, to enhance FH case identification in primary care [40]. The FAMCAT model may be more sensitive than DLCN, SBR or MEDPED [40] and is based on SBR criteria including family history details such as MI, FH, and raised cholesterol. However, its clinical utility has yet to be assessed.

There is emerging evidence that a large proportion of patients with a clinical diagnosis of FH in whom a causative mutation cannot be detected may have polygenic hypercholesterolaemia, i.e. an accumulation of mutations, each of which individually has a small LDL raising effect but which together result in the level of LDL cholesterol elevation typically found in FH patients [41]. By using a gene loading score based on 12 common LDL-raising Single Nucleotide Polymorphisms (SNPs), it has been shown that mutation negative FH cases demonstrate a significantly higher LDL gene loading score than in control subjects [41].

FH must also be differentiated from other dyslipidaemias such as Familial Combined Hyperlipidaemia (FCHL) and polygenic hypercholesterolaemia with increased Lp(a), both of which may be associated with increased vascular risk and may present with a clinical phenotype suggestive of FH. FCHL is a condition in which the patient has serum cholesterol and/or triglyceride concentration exceeding the 90th percentile of the age and sex matched healthy population and raised serum cholesterol and/or triglyceride in at least one first degree relative [42]. FCHL is considered to be the most common inherited lipid disorder and an important risk factor for vascular disease with a prevalence of 10–20 % amongst survivors of myocardial infarction. Although FCHL shows autosomal dominant inheritance with low penetration, the causative genes remain unclear. Associated with polygenic hypercholesterolaemia, Lp(a) is a circulating lipoprotein consisting of an LDL particle covalently bound to apolipoprotein(a). Circulating serum Lp(a) concentration is determined largely by variation in the apolipoprotein(a) gene and elevated Lp(a) is an independent risk factor for vascular disease [43]. Patients with polygenic hypercholesterolaemia and increased Lp(a) may therefore be clinically misdiagnosed as FH cases.

In order to diagnose FH, secondary causes of hyperlipidaemia must be ruled out by excluding cholestatic liver disease, hypothyroidism, significant proteinuria, diabetes mellitus and excess alcohol [3].

### Treatment

The National Institute for Health and Care Excellence (NICE) in the UK recommends FH cases should target a reduction in LDL-C levels of over 50 % from baseline (i.e. LDL-C levels before therapy) [44], whereas the European Society of Cardiology/European Atherosclerosis Society (EAS) recommends that the target level for acceptable LDL-C is <1.8 mmol/l in HeFH patients with confirmed CHD, and <2.5 mmol/l in HeFH patients without confirmed CHD [3].

Cohort comparisons [45] have demonstrated that HeFH patients treated with either simvastatin or atorvastatin had a 76 % overall risk reduction in CHD and no increased risk of adverse effects associated with statin therapy, including increased plasma liver enzyme activity, myalgia and, less commonly, rhabdomyolysis and myopathy [4]. However, high-intensity statin therapy may be associated with an increased risk of developing type 2 diabetes mellitus [46].

Almost 80 % of FH patients prescribed statin therapy do not attain the EAS recommended LDL-C levels [47]. In a small number of FH cases it has been suggested that this is due to statin resistance connected to polymorphisms in a number of genes, although this picture is currently unclear [48]. Alternative hypotheses include non-compliance due to adverse side-effects or patient choice [49].

Ezetimbe, a cholesterol lowering drug that blocks cholesterol absorption in the small intestine may be used in combination with statin therapy in patients who are not achieving lipid targets on statin monotherapy or in patients who are intolerant of statins. Other therapeutic agents include bile acid sequestrants, mipomersen (an inhibitor of apolipoprotein B-100 synthesis), lomitapide (a microsomal triglyceride transfer protein inhibitor), PCSK9 inhibitors [4], Fibrates [50] and Niacin [51], some of which have been demonstrated in combination with statins [52, 53]. In the case of HoFH, high-intensity statin treatment and LDL apheresis can be used [4].

It has been estimated that 96–98 % of CHD deaths in FH patients aged less than 40 years could potentially be averted with just statin therapy [54].

For children diagnosed with FH, lifestyle and diet are targeted. Many statins are approved for use from the ages of 8–10, although Atorvastatin has been approved for use from age 6 [18]. In the UK, NICE recommends consideration of statin therapy from age 10 years. Ezetimbe has been approved for use from the age of 10 in the USA and Europe and bile acid sequestrants have been approved for use from age 10 in the USA. Screening is recommended from the age of 5 although this can be complicated by issues around parental consent [18].

### Screening

FH has no formal disease classification under current WHO disease classifications [3, 55]. Cascade screening (CS), whereby family members are traced from an established FH index case, is more cost effective than any other screening strategy currently available [56] and is recommended in NICE guidelines [44]. Approximately half of the first degree relatives of an index case will be found to have the FH mutation [57]. NICE recommends against using SBR criteria for case detection of relatives of an index case as this results in under-diagnosis [44] and instead to use genetic testing (where the causative mutation in the index case has been identified) or to use age- and gender-specific LDL-C concentration where a genotypic result is not available in the index case [58].

At the introduction of CS in the Netherlands, 2039 relatives of 237 index cases were found to have FH with 39 % already taking treatment. A year later this had risen to 93 % [59]. To date, approximately 23,000 FH cases have been determined in the Netherlands by CS alone [17]. CS has also proven to be effective in Australia and Brazil, where each index case typically yields a further 2 cases [60, 61]. However, in the UK, it has proven less effective, yielding between 0.4 and 0.7 new cases per FH index case [57, 62, 63]. CS has still demonstrated its clinical utility in the UK [64], decreasing the age of FH diagnosis and increasing the number of people with FH on statin therapy [45].

It has been shown that even with systematic CS through to 3rd degree relatives, in a ‘best case’ detection scenario of 8.6 relatives per index case as in the Netherlands model [59], 17 % of FH cases must be identified as index cases in order to achieve a detection rate of 80 % of putative FH cases after introducing CS [65].

An alternative to CS is universal screening (UScr) in which a population is systematically screened. This has not yet been applied to FH, but could be undertaken by cholesterol measurement or genotyping in childhood. Genotyping might be more effective for populations in which founder effects occur with a restricted number of prevalent mutations. UScr has been a great public health triumph for detecting and treating disorders such as phenylketonuria (PKU), medium-chain acyl-CoA dehydrogenase deficiency (MCAD), cystic fibrosis (CF) [66] and cervical cancer [67]. The newborn screening programmes implemented for CF have shown direct benefits such as preventing malnutrition [68] and may lead to indirect benefits such as informing parental reproductive choices, reducing parental stress and facilitating clinical trial recruitment [69]. Such advantages have led some to advocate genome wide analyses from a single sample although there are significant ethical and regulatory considerations to doing so [70].

International guidelines advocate targeted screening for the identification of new FH index cases [71] in which screening can be directed at specific patient groups likely to show a high prevalence of FH such as those post acute coronary syndrome. A study based around an Australian coronary care unit demonstrated that the prevalence of possible/definite FH (as defined by DLCN criteria) was as high as 14.3 % in patients below the age of 60 with a current or prior history of coronary artery disease [72]. Such targeted screening is an effective strategy for identifying new FH index cases.

It has been suggested that new FH index cases could be identified systematically from electronic health records [73, 74], but a preliminary study yielded disappointing results with only 2 new definite FH index cases identified from a population of 12,100 [75]. However, a strategy that would likely yield a higher FH detection rate is Reverse Cascade Screening (RCS) [76]. This combines elements of UScr and CS and involves screening an infant’s total cholesterol (TC) when they receive vaccinations at 15 months of age. Following the identification of elevated cholesterol (here defined as 1.5 times the median for age), DNA analysis can be utilised to identify relevant mutations and, should they exist, the parents and grandparents would be tested subsequently. It has been suggested that by running this programme for one generation, most, if not all, FH cases would be detected and registered [65].

Screening has conventionally been undertaken by array or PCR amplification. Sanger sequencing has demonstrated its value, but has proven prohibitively expensive. However, Next Generation Sequencing (NGS) with its ability to undertake parallel sequencing relatively quickly, has shown great promise [77]. NGS has demonstrated high levels of specificity and sensitivity [78] in particular when combined with clinical criteria [79, 80].

The value of screening for FH mutations is not without controversy. As FH is only an indicator for likely elevated LDL-C and a proportion of elevated LDL-C cases are negative for the canonical variants, it has been argued that screening should focus on phenotype rather than genotype, both for the identification of index cases and in cascade screening, and that a focus on genetic screening can offer false reassurances to variant-negative patients who might still be at risk [81, 82]. The guidelines for screening of FH, provided by the National Lipid Association (USA), still focus principally on phenotypic diagnosis [83].

### Economics

Detection and treatment of FH leads to significant savings in healthcare costs [56]. In the UK, it is estimated that the identification and optimal treatment of all FH cases would save the NHS £380 million over a 55 year period, or £6.9 million/year [84]. When extrapolated to the EU, the savings would yield about €86 million per year [18]. NICE guidelines estimate that CS leads to an incremental cost effectiveness ratio (ICER) of £2,700 per quality adjusted life year (QALY). This intervention is considerably less expensive than the £20,000 to £30,000 per QALY ceiling that NICE defines as cost-effective [85]. Furthermore, the cost of FH testing is likely to drop by as much as four-fold with the introduction of next-generation sequencing [86] improving the cost effectiveness further. It has been estimated that CS and high-intensity statin therapy would lead to 101 fewer deaths/1000 FH patients by CHD [84].

### Discussion

UScr as a general population disease identification strategy has pitfalls in terms of cost and the role of unknown causative variants. However as sequencing costs continue to drop, it is likely that new mechanisms of action will be uncovered and doctors can protect the patient from the irresolution in the genomic data [87].

Several authors propose that the ideal screening scenario is the integration of CS and UScr strategies [2, 88]. Others posit that as the costs of disease management increases, while the cost of diagnostics decreases, UScr will become more cost-effective and attractive, rendering CS less attractive [89]. The hybrid proposal of RCS [76] may be more cost-effective than UScr and become more so if DNA sequencing continues to outpace Moore’s Law in terms of better, cheaper, faster performance [90, 91].

Early recognition of a child with FH, coupled with therapy from a young age, will impede, if not arrest, the onset of atherosclerosis [18]. UScr is already used for conditions such as phenylketonuria, which has a prevalence of 1 per 10,000, so the societal barriers to UScr should be lower for diseases such as FH where it has been demonstrated that testing and treating is clearly beneficial [89].

In the Netherlands, children with FH who have received counselling as part of their early intervention therapy have not taken up smoking, a lifestyle risk factor, in 100 % of cases. Additionally, these children have been shown to cope effectively with their diagnosis [18, 92].

## Conclusion

Familial Hypercholesterolaemia is a significant risk factor for cardiovascular disease, the leading cause of death globally. Familial Hypercholesterolaemia is an autosomal, dominant genetic disorder predominantly associated with pathogenic variants in the genes *LDLR*, *ApoB*, *LDLRAP1* and gain of function variants in *PCSK9*. Screening typically occurs using the Dutch Lipid Clinic Network, the Simon Broome Register or the Make Early Diagnosis to Prevent Early Death (MEDPED) criteria. Typically, a diagnosed case forms an index case from which a cascade screen is undertaken within the same family to identify as many new cases as possible. However, alternative screening programmes include systematic screens across a population (universal screening) and hybrid schemes in which cascade screening is applied to systematically screened subpopulations (reverse cascade screening). Cost-effectiveness is dependent on the scheme. Cascade screening in its typical form is highly cost-effective, although more systematic programmes may become more competitive if genome sequencing costs continue to fall.

The ability to identify FH patients at the earliest opportunity is both economically and socially beneficial with implications for mortality and morbidity.

Whichever screening strategy is optimal, studies of screening programmes will not only address FH as one of the world’s most prevalent and treatable inherited diseases, but have the potential to contribute to broader studies of hereditary diseases with similar traits.

## Abbreviations

ApoB: Apolipoprotein B
ARH: Autosomal Recessive Hypercholesterolaemia
CHD: Coronary Heart Disease
CS: Cascade Screening
DLCN: Dutch Lipid Clinic Network
EAS: European Atherosclerosis Society
ESC: European Society of Cardiology
FAMCAT: Familial Hypercholesterolaemia Case Ascertainment Tool
FCHL: Familial Combined Hyperlipidaemia
FH: Familial Hypercholesterolaemia
HDL: High Density Lipoprotein
HeFH: Heterozygous FH
HMGCoA: Hydroxymethylglutaryl coenzyme A
HoFH: Homozygous FH
ICER: Incremental Cost Effectiveness Ratio
IDL: Intermediate Density Lipoprotein
LDL: Low Density Lipoprotein
LDL-C: Low Density Lipoprotein Cholesterol
LDLR: Low Density Lipoprotein Receptor
LDLRAP1: Low Density Lipoprotein Receptor Adaptor Protein 1
LGY: Life Year Gained
LOVD: Leiden Open Variation Database
LP(a): Lipoprotein(a)
MEDPED: Make Early Diagnosis to Prevent Early Death
MI: Myocardial Infarction
NGS: Next Generation Sequencing
NHS: National Health Service (UK)
NICE: National Institute for Clinical Excellence
PCSK9: Protein Convertase Subtilison/Kexin Type 9
QALY: Quality Adjusted Life Year
RCS: Reverse Cascade Screening
SNP: Single Nucleotide Polymophism
TC: Total Cholesterol
UScr: Universal Screening
VLDL: Very Low Density Lipoprotein
WHO: World Health Organisation
